# Cellulose- and Saccharide-Based Orally Dispersible Thin Films Transform the Solid States and Dissolution Characteristics of Poorly Soluble Curcumin

**DOI:** 10.1155/2024/8596712

**Published:** 2024-05-27

**Authors:** Helmy Yusuf, Orchidea Meidy Nurintan Savitri, Nadia Natsya Al-Khalifi, Lavinia Gunawan, Brian Karno Chairul, M. Agus Syamsur Rijal, Dewi Isadiartuti, Retno Sari

**Affiliations:** Department of Pharmaceutical Sciences, Faculty of Pharmacy, Universitas Airlangga, Jl. Mulyorejo, Surabaya 60115, Indonesia

## Abstract

This study aimed at developing and optimizing the orally dispersible thin film (ODTF) containing a plant-derived drug—curcumin (CUR). CUR belongs to a biopharmaceutical classification system (BCS) class IV compound that requires improving its water solubility and tissue permeability preceding formulation. An ODTF was applied to produce a solid dispersion matrix for CUR to resolve such solubility and permeability problems. The film-forming polymers used in the study were cellulose-based (hydroxypropyl methylcellulose/HPMC and carboxymethylcellulose/CMC) and saccharide-based maltodextrin (MDX). Poloxamer (POL) was also employed as surfactant and solubilizer. The solvent casting technique was applied to produce the films. The ethanolic solution of CUR was mixed with an aqueous solution of POLs and then incorporated into different film-forming polymers prior to casting. The processing of the CUR with POL solution was intended to aid in the even dispersion of the drug in the polymeric matrices and enhance the wettability of the films. The physical state and properties of the films were characterized in terms of their morphology, crystallinity of the drug, and phase miscibility of the mixtures. The dissolution profile of the films was also evaluated in terms of dissolution rate and dissolution efficiency. The obtained ODTF products were smooth and flat-surfaced. Physical characterization also indicated that the CUR was homogeneously dispersed in the ODTFs and no longer existed as crystalline material as revealed by X-ray diffraction (XRD). The CUR was also not phase-separated from the films as disclosed by differential scanning calorimetry (DSC). Such dispersion was achieved through the solubilizing effect of POLs and compact polymeric film matrices that prevented the CUR from recrystallization. Furthermore, the ODTFs also improved the dissolution of CUR by 3.2-fold higher than the raw CUR. Overall, cellulose-based films had favorable physical properties compared with saccharide-based films.

## 1. Introduction

Alternative dosage forms for patients with swallowing difficulties such as pediatric and geriatric populations and other vulnerable patients are required to improve the acceptability and patients' adherence to the therapy [1, 2]. An ODTF dosage form suits such needs and offers an alternative route of administration as the drug is dissolved in the oral cavity and directly absorbed in the mucosal tissue into the systemic circulation. ODTFs have been reported to show rapid disintegration and fast-dissolving properties without the need for a large amount of liquid [3, 4].

ODTFs are thin films made of polymeric compounds that contain active pharmaceutical ingredients (APIs). The APIs can be entrapped in an amorphous form that enhances their solubility and stability [5–7]. ODTFs can be manufactured by solvent casting and hot-melt extrusion technique [8, 9]. The texture, the taste in the mouth, and the size affect the acceptability of ODTFs. Furthermore, the API solubility is the key factor that determines the size of ODTF as it affects the drug-loading capacity [10, 11]. However, a higher drug loading has an impact on the mechanical properties of the ODTF and a risk for recrystallization of the amorphous API [12, 13].

CUR is a plant-derived compound that is used to treat several diseases including neurodegenerative disorders and tuberculosis, which are still of high prevalence [14–16]. The therapeutic potencies of CUR have attracted many studies in aspects of its development into various delivery systems to bring it to clinical use [17, 18]. The major challenge is attributed to the poor solubility and permeability of CUR that is classified as class IV in the biopharmaceutical classification system [19, 20]. These drawbacks require solubility and permeability enhancements in the development of formulation. Several techniques have been used to improve the solubility of the CUR, including cyclodextrin complexation [21, 22], nanocrystals [22], or solid dispersion systems [23].

In the solid dispersion (SD) technique, hydrophilic polymers are used as matrices where the drug is molecularly dispersed in solid films. Such a system improves the drug's solubility and dissolution and leads to the enhancement of the bioavailability [6, 24]. The benefits are achieved through mechanisms including amorphization of crystalline drugs [24, 25], improvement of drugs' wettability [26, 27], and reduction in drugs' particle size. Many SD manufacturing techniques are relatively simple and low cost, including spray drying, supercritical fluid, hot-melt extrusion, and solvent evaporation [28, 29].

To improve the solubility of lipophilic drugs in hydrophilic matrices, surfactants are often used as solubilizing agents. Many types of surfactants, e.g., anionic, zwitterionic, and nonionic surfactants, were used to disrupt polymeric nanoparticles in drug analysis [30]; however, the use of nonionic surfactants such as poloxamers is beneficial for preparing drug-containing solid dispersions in the form of ODTFs. Among surfactants that are used as solubilizing agents, poloxamer has been one of the most attractive surfactants being studied [31]. Poloxamer fits the purpose of being a solubilizing surfactant. The amphiphilic properties of poloxamer are due to its molecular structure that consists of a hydrophobic group of polypropylene oxide (PPO) that is bound to two hydrophilic chains of polyethylene oxide (PEO) and forms micelles in an aqueous environment that solubilizes hydrophobic drugs [31, 32].

The present study aimed to develop and evaluate the physical properties of CUR solid dispersion in the form of ODTFs. We investigated the physical properties of different film-forming polymers with the inclusion of POLs as solubilizing agents using the technique of solvent evaporation casting. Those properties include morphology determined by scanning electron microscopy (SEM), the crystallinity of the entrapped CUR determined by X-ray diffractometry (XRD), the thermal properties of the ODTFs determined by differential scanning calorimetry (DSC), and the dissolution profile of the developed ODTFs. The effect of HPMC, NaCMC, and MDX as film-forming polymers on those properties was evaluated.

## 2. Materials and Methods

### 2.1. Materials

Curcumin, poloxamer 188 (Kolliphor® P 188), poloxamer 407 (Pluronic® F-127), sodium carboxymethyl cellulose (NaCMC), maltodextrin, and ethanol at analytical grade were purchased from Sigma-Aldrich (Singapore). Hydroxypropyl methylcellulose (METOLOSE®) was purchased from Shin-Etsu (Japan).

### 2.2. Preparation of ODTFs

CUR was dissolved in ethanol and mixed with POL aqueous solution to initiate a miscible liquid mixture. A polymer mixture at serial concentrations was prepared to accommodate their film-forming capacities. The polymer was dissolved in distilled water under constant and vigorous stirring at 500 rpm at room temperature, using a magnetic stirrer. The CUR- and POL-containing mixture solution was added dropwise to the mixture of polymer solutions at the fixed ratios and stirred constantly at 500 rpm for 1 h at room temperature. The resulting solution was used to cast CUR-loaded ODTFs. The CUR-loaded mixtures were poured into a flat-bottomed chamber with a thickness of 1000 *μ*m and dried at room temperature. Next, the resultant films were cut into rectangular strips (3 × 4 cm) and stored at 4–8°C prior to analysis. Prepared formulations were weighed as presented in Table 1.

### 2.3. Scanning Electron Microscopy (SEM)

SEM (Philips X series, The Netherlands) was applied to observe the morphology of CUR-loaded ODTFs. The ODTF samples and the individual pure materials were dropped and fixed to the metal stub. Next, samples were gold coated and vacuumed by a sputter coater at a setting condition of 10 mA for 20 s. Morphologies of the samples were captured at 15 kV.

### 2.4. X-Ray Diffractometry (XRD)

The XRD (Philips X'Pert PRO; Panalytical, The Netherlands) was used to analyze the crystalline structure of the pure materials and the ODTFs containing nanoparticles. The diffraction patterns were recorded at conditions of 25 mA and 40 kV over the 2*θ* range of 10° to 45° at a rate of 2°/min step.

### 2.5. Differential Scanning Calorimetry (DSC)

The thermodynamic properties of pure materials and prepared ODTFs were examined using DSC (Mettler-Toledo, Switzerland). Weighed samples at an amount of 2–8 mg were put in an aluminum crucible pan, and the lid was crimped to seal the pan. It was heated at a heating rate of 10°C/min within the range of 30°C and 240°C. Thermodynamic events were determined at the top of the peak.

### 2.6. Dissolution Study

The dissolution studies were performed to investigate the release profile of CUR from the ODTFs using a type 2 USP paddle dissolution apparatus (Erweka, Germany). The dissolution medium was 500 mL of phosphate-buffered saline pH 6.8 at 37 ± 1°C and maintained under sink conditions. A paddle rotation speed of 50 rpm was applied during the study. ODTFs equivalent to 1 mg of CUR were weighed carefully based on the prior drug content analysis using spectrophotometry. The raw CUR was also weighed at 1 mg for the dissolution test. The selected amount was based on the precalculated sink condition maintained. Samples were collected in 5 ml at 5, 10, 15, 30, 45, 60, and 75 min and replaced with the same amount of medium. The collected samples were filtered through a 0.22 *μ*m Millipore® filter immediately prior to spectrophotometry analysis. The amount of dissolved CUR in the dissolution medium was determined at *λ*=430 nm (spectrophotometer; Shimadzu, Japan). Dissolution was performed in triplicate for each sample to calculate the drug-release profile. General dissolution parameters, i.e., dissolution rate (DR) and dissolution efficiency (DE), were calculated based on the following equations [33, 34]:

(1) $$\begin{array}{c} \text{DR}=\frac{dC}{dt}=k\left(\text{Cs}-\text{Ct}\right), \end{array}$$

where d*C*/d*t* is the dissolution rate, *k* is the constant of dissolution rate, Cs is the concentration of the drug in the immediacy of dissolving or drug solubility, and Ct is the concentration of the dissolved drug at time *t*.

(2) $$\begin{array}{c} \text{DE}=\frac{\int_{t1}^{t2}y.\;dt}{y100.\;\left(t2-t1\right)}\times 100\%, \end{array}$$

where *y* is the percentage of dissolved drugs. DE is the area under the curve between *t*1 and *t*2 time points denoted as a percentage of the curve at maximum dissolution, and *y*100 is the percentage of dissolved drugs over the same time considered 100%.

### 2.7. Statistical Analysis

The software GraphPad® InStat (San Diego, CA) was used to conduct the tests. Using a parametric one-way ANOVA test, the dissolution experimental data were examined. Statistical significance was determined using 95% confidence limits.

## 3. Results and Discussion

### 3.1. Morphology

**Figure 1:**
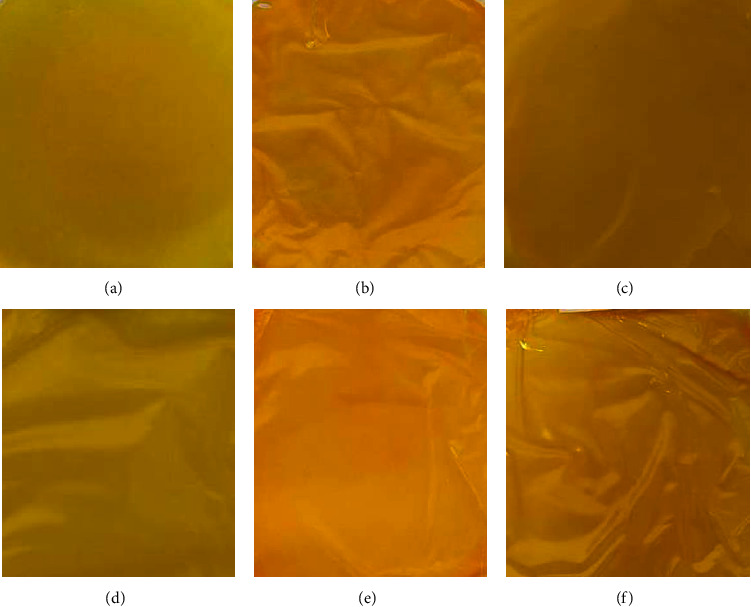
Organoleptic images of all developed ODTF formulations prepared according to Table 1 by the casting method: (a) ODTF-1, (b) ODTF-2, (c) ODTF-3, (d) ODTF-4, (e) ODTF-5, and (f) ODTF-6.

A visual evaluation was carried out to disclose the effects of drug loads and film-forming polymers on the organoleptic appearance of the ODTFs. Different drug-loading and film-forming polymers were applied to the developed formulations (Table 1). The visual inspection showed that all the ODTFs' surfaces were smooth and homogeneous yellow transparent sheets. The colours were slightly similar regardless of the exerting different drug loadings and types of polymers (see Figure 1). Organoleptically, all the films were flat-surfaced without any wrinkles. All of them were easily detachable from the cast. The employed casting technique successfully produced smooth-surfaced films without entrapped air bubbles [35, 36]. The ODTF texture has an impact on its acceptability, and the film roughness is significantly affected by insoluble drug particles [37]. In this case, all materials were completely dissolved in the solvents and not phase-separated upon drying.

**Figure 2:**
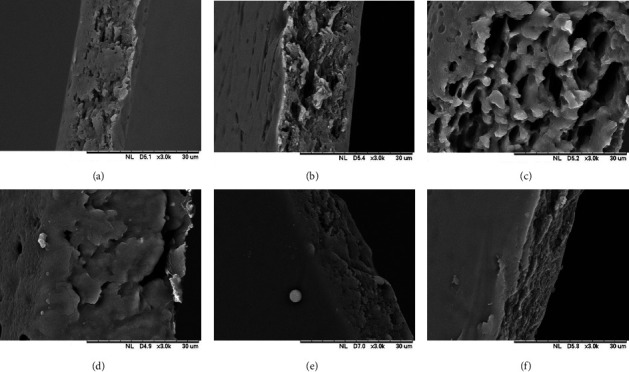
SEM Images of the longitudinal cross section of all developed ODTF formulations prepared according to Table 1 by the casting method: (a) ODTF-1, (b) ODTF-2, (c) ODTF-3, (d) ODTF-4, (e) ODTF-5, and (f) ODTF-6.

SEM images of the surfaces of all the films were similar, i.e., smooth and flat (data not shown). Hence, SEM was employed to evaluate the microsized longitudinal slice of the films to get more information. As shown in Figure 2, SEM images revealed vague differences in the morphologies of the films. Insoluble particles were not detected, indicating that the CUR was completely dissolved in the film matrix and may not stance a problem. Nevertheless, there were a few microsized cavities within the films that can be attributed to the CUR solubilization process using ethanol. During the solubilization process, agitation was employed using a stirrer, which might have trapped tiny air bubbles beneath the surface and later diffused to the outer layer, resulting in a small cavitation and porous layer [38]. The ODTF-3 and ODTF-4 using a combination of HPMC and MDX as film-forming polymers showed more cavities in the structure. This can be explained by previously reported studies in regards to the use of MDX where the pore size of freeze-dried films can be affected by the nature molecular weight of MDX [39, 40].

### 3.2. DSC Analysis

**Figure 3:**
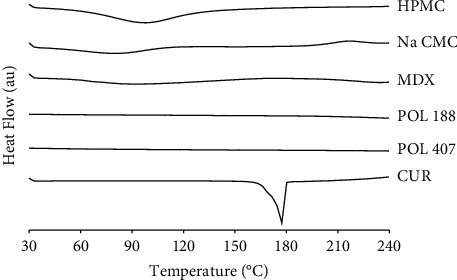
DSC thermograms of raw materials: hydroxypropyl methylcellulose (HPMC); sodium carboxymethyl cellulose (NaCMC); maltodextrin (MDX); poloxamer 188 (POL 188); poloxamer 407 (POL 407); curcumin (CUR).

DSC analysis was employed to characterize each individual raw component and its multicomponent mixtures in the developed formulations. The analysis is robust and fast in screening the compatibility of the excipients with either among excipients or the drug with excipients. With only a small-sized sample, the compatibility evaluation can be performed in a very quick and simple procedure. This makes this technique very advantageous, especially for the initial stage of the ODTF formulation development. The DSC thermogram of the CUR and all excipients is presented in Figure 3.

As shown in Figure 3, an endothermic event observed at 174°C corresponded to the CUR melting temperature (Tm), which was similar to that reported previously [17]. Such a sharp endothermic peak concluded that the raw CUR is a crystalline material. Next, the HPMC thermogram showed a very broad low-temperature endothermic peak with the onset peak started at 40°C expanded to 130°C. As the peak was broad, a conventional DSC technique was not able to determine whether it was a glass transition or melting event. However, as the HPMC is an amorphous polymer, most see such a broad peak as a glass transition event as reported by others using the MTDSC technique [41]. Similar states were observed for the other raw materials, e.g., NaCMC, MDX, POL 188, and POL 407, as they are also noncrystalline structures as denoted by the absence of endothermic peaks.

**Figure 4:**
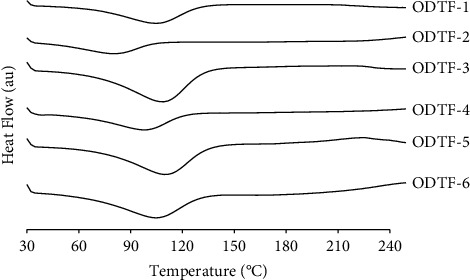
DSC thermograms of all developed ODTF formulations prepared according to Table 1 by the casting method.

The thermograms of all the cast ODTFs are presented in Figure 4. The results suggest that the crystalline CUR in all developed ODTFs was transformed into a full amorphous state as denoted by the disappearance of the previously observed endothermic peak of CUR, as observed in Figure 3. In the case of the ODTF-2 that is composed of HPMC alone, the thermodynamic event is visible at a lower temperature, i.e., a broad endothermic peak with onset at 40°C to 110°C corresponding to the dominant phase transition of HPMC because of the exclusion of NaCMC and MDX in the ODTF-2. In contrast, the incorporations of the two film-forming components, both NaCMC and MDX, in the formulations have shifted the broad peak to higher temperatures at c.a. 60°C to 140°C. The initiation of phase transition started at 60°C and ended at 140°C and was observed for the formulations consisting of a polymer mixture of HPMC with both MDX (ODTF-3 and ODTF-4) and NaCMC (ODTF-5 and ODTF-6). It was suggested that the event may have occurred due to intensified interactions among the film components. Despite such complexity, very modest curve profiles were observed, which may be associated with the optimal interaction among the components.

### 3.3. XRD Analysis

**Figure 5:**
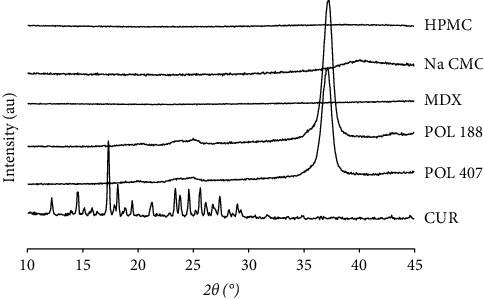
XRD diffractograms of raw materials: hydroxypropyl methylcellulose (HPMC); sodium carboxymethyl cellulose (NaCMC); maltodextrin (MDX); poloxamer 188 (POL 188); poloxamer 407 (POL 407); curcumin (CUR).

All raw materials and the internal structure of CUR crystals incorporated in the prepared ODTFs were investigated for possible potential changes using XRD. As presented in Figure 5, XRD analysis demonstrated that the raw CUR was crystalline and in agreement with DSC data, as indicated by very intensive and sharp peaks in the diffractogram. HPMC, NaCMC, and MDX were amorphous materials, as indicated by flat without distinguished sharp peaks pattern in the diffractograms. POL 188 and POL 407 exhibited an identical single sharp peak due to their crystal-like nature [24].

**Figure 6:**
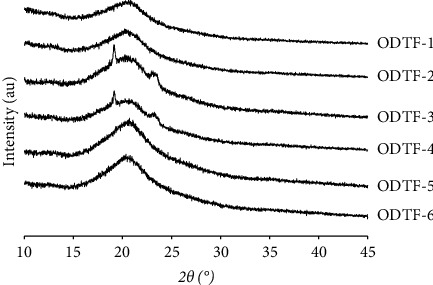
XRD diffractograms of all developed ODTF formulations prepared according to Table 1 by the casting method.

The crystalline CUR shown in Figure 5 was no longer observed in all ODTFs containing CUR, indicating that CUR was no longer crystalline in the formulations (Figure 6). There were no apparent diffraction patterns among ODTF formulations, indicating that the CUR was preserved in an amorphous state regardless of the increased concentration of CUR in the films. Such an amorphous state was expected to enhance the dissolution and bioavailability of the CUR. These XRD analysis data suggested that CUR in the ODTFs agreed with the DSC data. As such, it should facilitate a much higher percentage of dissolved CUR compared with the raw CUR.

The diffractograms of all ODTFs containing CUR indicate that the drug undertakes amorphization, regardless of the increased CUR concentration, as indicated by broad amorphous patterns (Figure 6). Despite the diffractograms of ODTF-3 and ODTF-4 films designated amorphization of the samples, it is worth noting that they also exposed a single small peak that looked like a small crystalline fraction. However, as the X-ray beam interacts with only a small spot on the sample surface, a small portion of crystalline material may be hit by the beam. Even a small portion of the contaminant might also cause similar results. Such an incident has been considered as one of the XRD limitations. Nevertheless, the whole diffractogram showed an amorphous pattern, and this small peak could be disregarded. This was confirmed by the DSC data, where a visible endothermic peak of CUR was not detected in the thermograms, indicating the absence of a crystalline phase for the CUR. Thus, it might be concluded that the CUR underwent amorphization.

### 3.4. Dissolution Study

**Figure 7:**
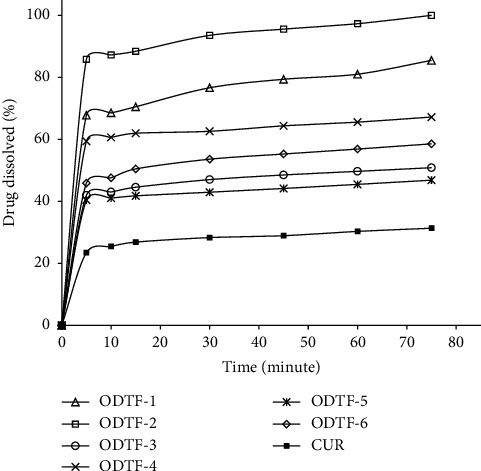
Dissolution profile of the raw drug and the formulation in ODTFs: curcumin (CUR), all developed ODTF formulations (ODTF-1 to ODTF-6) prepared according to Table 1 by the casting method.

CUR is a weak base and poorly water-soluble compound. Despite the weak base compounds being more soluble at lower pH, a phosphate-buffered saline pH 6.8 was used in the dissolution study and was still able to provide a sink condition for the determination of the dissolution behavior. The dissolution studies showed significant effects of film-forming polymers and poloxamers on the drug release from the formulations. The percentages of dissolved CUR from all ODTFs were significantly higher than those of the raw CUR (see Figure 7). The increase varied among formulations where HPMC as a film-forming polymer was superior to NaCMC and MDX. The overall increase in the percentage of drug dissolution was significant among formulations (*p* < 0.05). In respective order, the increases from the highest to the lowest were as follows: ODTF-2 > ODTF-1 > ODTF-4 > ODTF-6 > ODTF-3 > ODTF-5, ranging from 1.5 to 3.2 times higher than the raw CUR.

The improvement in CUR dissolution was related to many factors. First, poloxamer 188 and poloxamer 407 might facilitate the wetting of the drug. The improved wettability of ODTFs then facilitated water penetration into the polymeric lattice and dissolved the drug [42]. Secondly, poloxamer 188 and poloxamer 407 were also able to form micelles above their critical micelle concertation (CMC), which facilitated the solubilization of the drug. Poloxamers contain a hydrophobic PPO chain group in the inner part—where the hydrophobic drug is entrapped—whilst hydrophilic PEO chains at the outer part of the micelle facilitate the interface with the aqueous environment. As hydrophobic CUR molecules are present in the poloxamer solution above CMC, they spontaneously subset between the micellar and aqueous phases. In this process, reduced free energy results in the formation of solution that is thermodynamically stable [43]. This way, the CUR solubility was improved. In each formulation that used the same polymer matrix, employing a higher concentration of CUR resulted in a higher percentage of the dissolved drug than in the formulation with a lower concentration. Hence, a higher concentration is worth further development.

**Figure 8:**
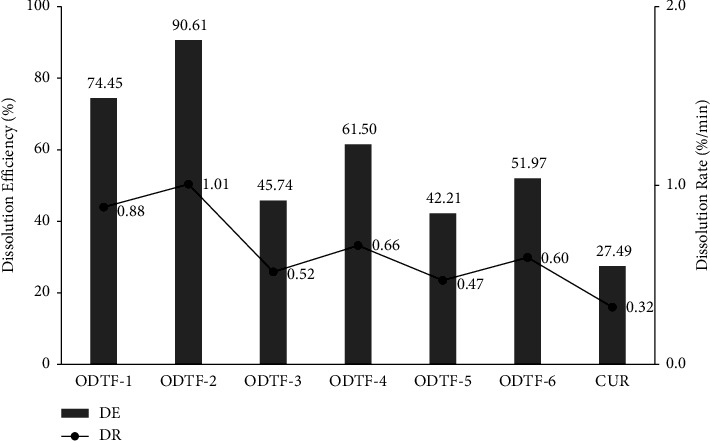
Dissolution's general parameters (DE and DR) of raw drug and the formulation in ODTFs: curcumin (CUR), all developed ODTF formulations (ODTF-1 to ODTF-6) prepared according to Table 1 by the casting method.

The dissolution profile of CUR from the developed ODTFs was significantly improved compared with the raw CUR (Figure 8). All the prepared ODTFs exhibited DR and DE, which were in the same respective order from the highest to the lowest, as follows: ODTF-2 > ODTF-1 > ODTF-4 > ODTF-6 > ODTF-3 > ODTF-5 with increases of 3.2-fold, 2.8-fold, 2.1-fold, 1.9-fold, 1.6-fold, and 1.5-fold in the respective order. These dissolution efficiencies (DEs) of the ODTFs were significantly higher than those of the raw CUR (*p* < 0.05), whilst the dissolution rates (DRs) were not significantly different (*p* > 0.05). Nevertheless, the DR and DE of ODTF-3 and ODTF-5 were noticeably lower than the rest of the formulations with only 1.6-fold and 1.5-fold, respectively. The ODTF-3 used a mixture of MDX and HPMC, whilst the ODTF-5 used a mixture of NaCMC and HPMC where both formulations included a lower amount of CUR. It might be assumed that such mixture polymers have been a barrier to the release of CUR with a lower concentration. The low concentration of CUR has been a factor in terms of capability to initiate diffusion from the matrix, which resulted in a relatively lower dissolution. Despite such a mixture forming a more compact matrix, the low concentration of CUR has been deterring dissolution compared with formulations with higher concentrations of CUR. Henceforth, in the case of employing a lower CUR concentration, both amorphization of CUR and the presence of the hydrophilic polymers, i.e., HPMC, NaCMC, MDX, and POLs in the formulations, might not fully facilitate diffusion of the CUR and its release.

## 4. Conclusions

The development of ODTFs containing poorly water-soluble CUR in the form of solid dispersions using POLs with HPMC, NaCMC, and/or MDX matrices showed enhanced properties as compared to raw CUR. This can be attributed to the solubilization of CUR molecules inside nanoaggregates formed by the amphiphilic POLs. Further incorporation of the soluble CUR into the film matrices led to an improvement in its amorphization and an increase in dissolution. The type of polymer affects the dissolution of CUR from the films where HPMC and NaCMC-based films exhibited better dissolution than the MDX-based film. A faster release rate was observed for the films with HPMC and CMC-based formulae. Among the three polymers used as a film base, HPMC had favorable physical properties and the amount of dissolved CUR. To conclude, the present study showed that the incorporation of CUR into ODTFs provides several advantages including improved dissolution profile and enhanced physical properties. It is worth investigating further developments, evaluations of biological activities, and antioxidant and phenolic compound tests for the developed ODTFs.

## Figures and Tables

**Table 1:** Composition of ODTF formulations.

Formulation	Content of film-forming components (% w/w)					
	CUR	HPMC	NaCMC	MDX	POL 188	POL 407
ODTF-1	1	86	—	—	8.5	4.5
ODTF-2	2	85	—	—	8.5	4.5
ODTF-3	1	65	—	21	8.5	4.5
ODTF-4	2	64	—	21	8.5	4.5
ODTF-5	1	65	21	—	8.5	4.5
ODTF-6	2	64	21	—	8.5	4.5

## Data Availability

The data used to support the findings of this study are available from the corresponding author upon reasonable request.
